# Comparative Evaluation Between Conventional Earplugs and Modified Earplugs in Reducing the Severity of Gag Reflex While Making Impression in Completely Edentulous Patients: A Randomized Controlled Trial

**DOI:** 10.7759/cureus.70637

**Published:** 2024-10-01

**Authors:** Ritul Jain, Sweta G Pisulkar, Akansha Bansod, Arushi Beri, Shruti Deshmukh

**Affiliations:** 1 Department of Prosthodontics and Crown and Bridge, Sharad Pawar Dental College and Hospital, Datta Meghe Institute of Higher Education and Research, Wardha, IND

**Keywords:** dental impression technique, earplug, edentulous patients, gag reflex, music-supported therapy

## Abstract

Background

The gag reflex is a common problem encountered during impression-making in completely edentulous patients. The use of music therapy and earplugs reduces gag reflex severity. However, no comparative study has been reported to establish their effectiveness.

Methods

This randomized controlled trial randomly assigns 56 completely edentulous patients with gag reflexes to either the conventional earplug group or modified earplug group. The patients will, with earplugs on, listen to music while waiting and resting for a period of 10 minutes before maxillary impression-making. The Gagging Severity Index (GSI) and Gagging Prevention Index (GPI) will be measured to assess whether music therapy with earplugs would be effective in minimizing the gag reflex.

Results

The results showed that, in the conventional earplug group, GPI at 2.9643 ± 1.20130 was more effective than GSI at 2.9286 ± 0.81325, but it was not significant at p = 0.051. On the contrary, in the modified earplug group, a gain was observed with respect to the significant effectiveness of GPI at 2.8929 ± 1.31485 compared to GSI at 2.6786 ± 0.77237, reaching a significance of p = 0.003. The result also indicates no significant difference between the two groups in GPI at p = 0.430.

Conclusion

There was a reduction in the severity of the gag reflex with the use of modified earplugs as compared to a conventional earplug. Music therapy and earplugs can be used as an adjunct to reduce gag reflex during impression-making in completely edentulous patients.

## Introduction

The most common problem dentists face in clinical practice is gagging. The protective reflex of gagging is to prevent the foreign material from dropping into the respiratory system. Due to such heightened sensitivity, the patients cannot tolerate any kind of foreign object on or in their mouth, thereby making the situation a very distressing experience for the patient and the dentist alike. Certain diagnostic procedures may become laborious and time-consuming in the process of completing treatment. The genesis of gagging is complicated. Patients may gag due to systemic illnesses, anatomical reasons, or psychological abnormalities. The literature has discussed a number of therapy approaches, including behavioral strategies, acupressure, hypnosis, acupuncture, systematic desensitization, and pharmaceutical management [1].

It has been indicated that auricular point acupuncture works in treating severe gag reflexes. Similarly, Hegus point acupuncture has also been associated with similar findings [2]. The major afferent pathway for the gag reflex is assumed to be the glossopharyngeal nerve that provides sensation to the mucosa of the oral cavity. The gag reflex or, more correctly, the pharyngeal reflex is a protective reflex which prevents food from entering the airway. It is essentially initiated by the tactile stimulation of the mucosa in the oropharynx. This involves regions such as the root of the tongue, soft palate, and anterior pillars of the fauces. These regions are supplied by the trigeminal nerve or cranial nerve V and also play an important role in the eliciting of the gag reflex.

The gag reflex may be intact even in the case of damage to the glossopharyngeal nerve, which is the cranial nerve IX. This persistence is due to the compensatory activity of the trigeminal nerve for the deficit provided by the glossopharyngeal nerve. As in other protective reflexes, neural pathways in this reflex have redundant and partial overlap so that this may serve as a critical protective function for the airway. These connections indicate the extent of the neural network involved in incorporating sensory input from both the external ear and oropharyngeal region, emphasizing coordination for reflexive and sensory responses in these regions [3].

All these sensations have their convergence at the spinal tract nucleus of the trigeminal nerve in the brainstem. The recent literature reports that the oropharyngeal regions and ear skin signals may converge at various anatomical levels including the cortex and brainstem. Malignancies of oral, pharyngeal, and laryngeal inlet have been documented to cause referred discomfort over the epidermis of the esophageal canopy [4]. Referred otalgia due to disorders of the maxillofacial region is an identifiable cause. This occurs because the course of the trigeminal nerve, innervating the face and jaw, runs with the nerves carrying pain to the ear. Therefore, disorders that might seem as far from your ears as dental problems or sinusitis give rise to ear pain on their own without any injury to the ear at all. For this reason, identifying such a pattern helps in the correct diagnosis and treatment of referred otalgia [2,5].

The anti-gagging point of the ear is on the outer skin layer of the external acoustic meatus. This area is supplied by the auricular branch of the vagus nerve, while the skin overlying that part that is closest to the auricle is supplied by the auriculotemporal branch of the trigeminal nerve, itself a branch of the mandibular division. Branches of the vagus and trigeminal nerves determine the sensory and motor functions of the region of the larynx, pharynx, and palate. It has been proposed that the activation of the auricular acupuncture point, which prevents the gag reflex, may reduce mylohyoid muscle contraction [4,5].

The present study will evaluate the efficacy of low-cost, widely available earplugs with music and modified earplugs compressing the relaxation point on the external ear to stimulate the external ear and suppress the severe gag reflex brought about by impression-taking of the maxillary teeth in a completely edentulous patient.

## Materials and methods

Study design and setting

This randomized controlled trial was conducted in the hospital setting, within the Department of Prosthodontics at Sharad Pawar Dental College, Sawangi (Meghe), Wardha, with an IEC reference number of IEC/2023/861.

Selection of participants

Fifty-six patients who had gag reflexes with complete edentulism were selected from the Outpatient Department (OPD) of the Department of Prosthodontics. Written informed consent was sought from each patient prior to the trial, and all participants were ensured of confidentiality and anonymity. The patients were recruited based on a set of predefined inclusion and exclusion criteria. The inclusion criteria for this study were edentulous patients having gagging reflexes with a Gagging Severity Index of II or III (GSII or GSIII), patients aged 25-99 years, and patients who consented and were willing to participate. The exclusion criteria were unwillingness to agree and/or participate and maxillary defects.

Sample size calculation

The sample size was calculated based on the effect of earplugs in reducing gag reflex from a previous study [3], which was reported to be 90%. Assuming a 20% clinically significant superiority, the sample size was calculated to be 28 per group, amounting to a total sample size of 56. This calculation was done using a statistical software package (Raosoft online sample size calculator, Raosoft Inc., Seattle, WA, USA), with a significance level of 0.05 and a power of 0.8. 

Study procedure

**Figure 1:**
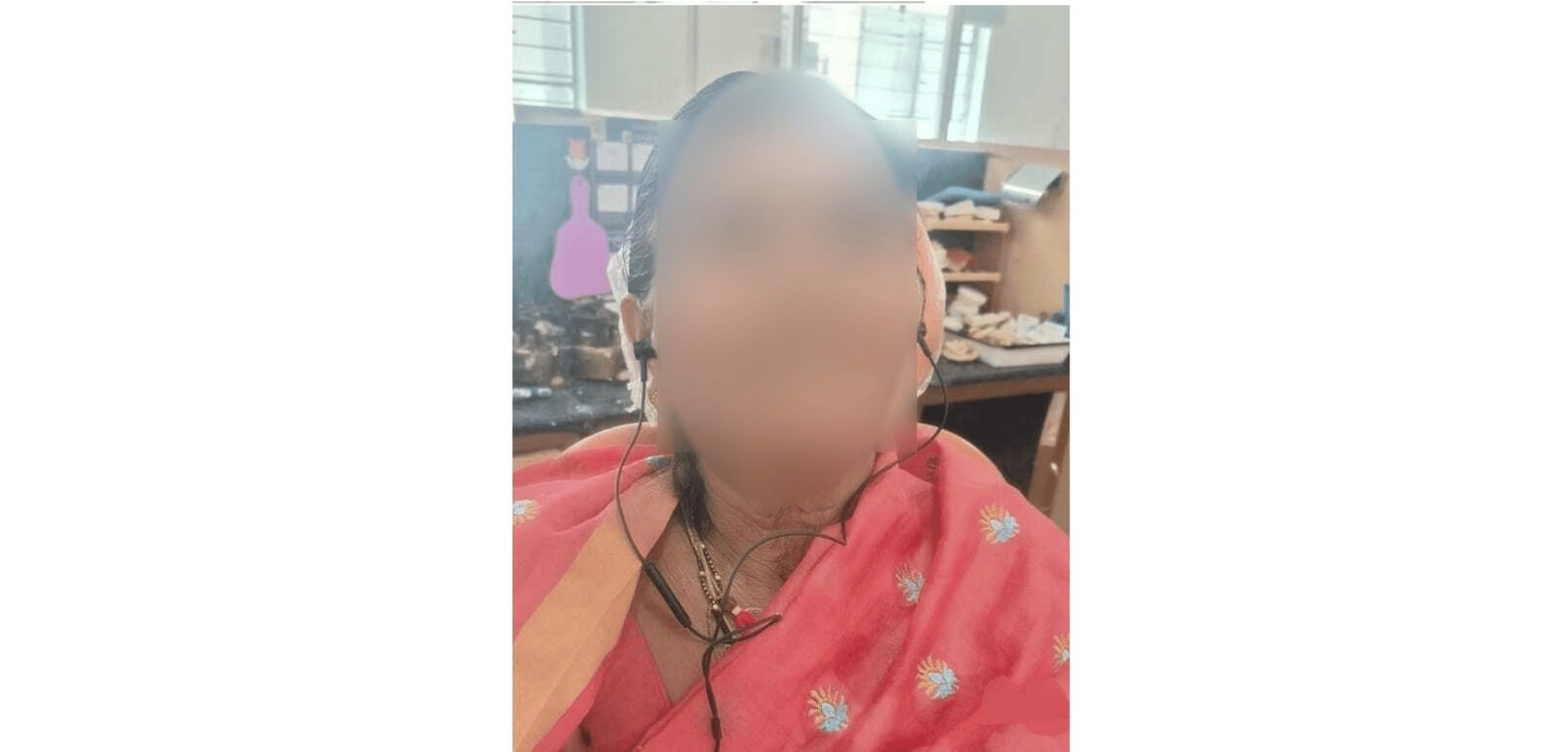
Conventional earplugs utilized for the study

Patients were allowed to sit in the waiting room and listened to music through earplugs for a period of 10 minutes to acclimate themselves to the environment. Maxillary impression was obtained using Zhermack alginate impression material (Rovigo, Italy). Patients were randomly assigned to either the conventional earplug group or the modified earplug group. The conventional earplugs were the boAt Rockerz 255 Pro+ Bluetooth Neckband earplugs, manufactured by boAt (Mumbai, India). The modified earplugs were also the boAt Rockerz 255 Pro+ Bluetooth Neckband earplugs, but we designed the modified earplugs to have a unique ergonomic shape that provided a more comfortable and snug fit in the ear canal. This design feature allowed for better sound isolation and reduced external stimuli, enabling patients to relax more easily and reducing anxiety and stress, common triggers of the gag reflex. GSI and GPI were employed to measure the music therapy's effectiveness and that of the use of earplugs in gag minimization. The GSI graded the gagging reflex of the patients as very mild, mild, moderate, severe, or very severe. Figure 1 depicts the conventional earplug group and the patient employed in this group.

**Figure 2:**
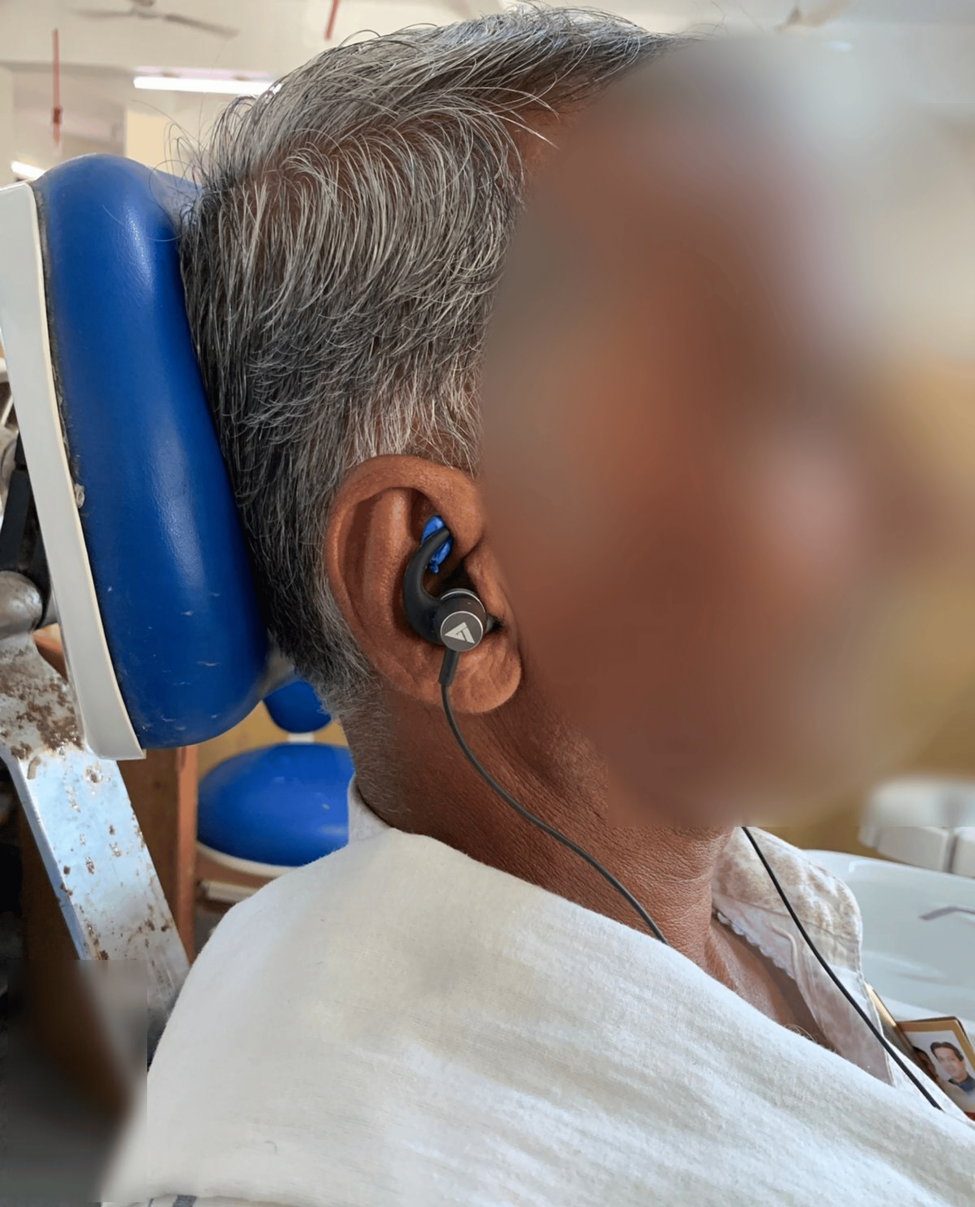
Modified earplugs in the patient

Figure 2 shows the modified earplug group and the patient utilized in this group.

Gag was measured as a result of the treatment by the use of the GPI, which ranged from obtunded gag reflex to severe gag reflex [5].

Statistical analysis

Data analyses involved the use of unpaired t-tests in comparing the means of GSI and GPI between the conventional earplug group and the modified earplug group. The statistical analysis was performed using the IBM SPSS Statistics for Windows, V. 26.0 (IBM Corp., Armonk, NY, USA), with a significance level of 0.05. The results were presented as mean ± standard deviation, and the confidence interval was set at 95%.

## Results

Table 1 shows the comparison of the GSI and GPI of normal earplugs using an unpaired t-test. From this table, we conclude that in the case of normal earplugs, GSI (2.9286 ± 0.81325) was found to be less effective than GPI (2.9643 ± 1.20130); this association was found to be statistically insignificant (p = 0.051).

**Table 1:** Comparison of the GSI and GPI of normal earplugs using unpaired t-test GSI: Gagging Severity Index; GPI: Gagging Preventive Index; N: sample size; Mean: mean; Std. deviation: standard deviation; Std. error mean: standard error of the mean; T-value: t-statistic; P-value: probability value

Variables	N	Mean	Std. deviation	Std. error mean	T-value	P-value
GSI	28	2.9286	0.81325	0.15369	0.130	0.051
GPI	28	2.9643	1.20130	0.22702		

Table 2 shows the comparison of the GSI and GPI of modified earplugs using an unpaired t-test. In the case of modified earplugs, GSI (2.6786 ± 0.77237) was found to be less effective than GPI (2.8929 ± 1.31485); this association was found to be statistically significant (p = 0.003).

**Table 2:** Comparison of the GSI and GPI of modified earplugs using unpaired t-test GSI: Gagging Severity Index; GPI: Gagging Preventive Index; N: sample size; Mean: mean; Std. deviation: standard deviation; Std. error mean: standard error of the mean; T-value: t-statistic; P-value: probability value

Variables	N	Mean	Std. deviation	Std. error mean	T-value	P-value
GSI	28	2.6786	0.77237	0.14596	0.744	0.003
GPI	28	2.8929	1.31485	0.24848		

Table 3 shows the comparison of the GPI of normal and modified earplugs using an unpaired t-test and shows the GPI of normal earplugs (2.9643 ± 1.20130) was found to be less effective than the GPI of modified earplugs (2.8929 ± 1.31485); this association was found to be statistically insignificant (p = 0.430).

**Table 3:** Comparison of the GPI of normal and modified earplugs using unpaired t-test GSI: Gagging Severity Index; GPI: Gagging Preventive Index; N: sample size; Mean: mean; Std. deviation: standard deviation; Std. error mean: standard error of the mean; T-value: t-statistic; P-value: probability value

Normal	N	Mean	Std. deviation	Std. error mean	T-value	P-value
Normal	28	2.9643	1.20130	0.22702	0.212	0.430
Modified	28	2.8929	1.31485	0.24848		

## Discussion

Our obtained assessments suggest that, relative to the standard earplugs, the adapted earplugs constitute a more effective music therapy adjunct in reducing gag reflex severity. This has important therapeutic implications in view of the fact that it provides a rapid, non-invasive way of stopping gauging when an impression is taken, thus making patients comfortable and compliant. These findings also contribute to the literature on music therapy and earplugs as a gag reflex reduction modality, underlining potential benefits for this most common of problems from a multimodal approach. The optimal design and mechanism of earplugs in gag reflex reduction and evaluation of the success of this intervention across demographics are some areas that may be considered in future research.

Comparing these results with previous studies, we find several similarities and differences. First, our results of the study on the efficacy of earplugs in lessening the severity of gag reflex are in line with the study from Cakmak et al. [3], which stated that earplugs can be useful in avoiding the gag reflex during procedures such as taking maxillary tooth impressions. Additionally, the results of our investigation on the benefits of music therapy in decreasing the intensity of the gag reflex support the information presented by Mustafa et al. [1], who noted that a musical intervention could enhance occupational settings and reduce symptoms of gagging and anxiety in patients.

Our findings, however, disagree with those of Gupta et al. [6], who created a hypothesis which involved the anterior meatus region in the distribution of Arnold's nerve but was not the focus of our investigation. We also did not investigate the relationship involving ear acupuncture to treat anxiety reduction as investigated by Wang et al. [7]. Moreover, unlike one other research [8], ours did not take into account the topography of the auriculotemporal nerve and the effect on pain localization in the masticatory system and face areas.

In the latter part of our findings, that is, comparing the GPI of regular and modified earplugs, results are novel and provide further details on the effectiveness of earplugs in reducing gag reflex intensity. By contrast, most of the available literature compares no more than one kind of earplugs or modification to the use of music therapy or earplugs alone as an intervention.

It is also put forward in our study that GPI is more effective in reducing the severity of gag reflex than GSI, especially with custom-made earplugs. This agrees with a study conducted by Agrawal et al. [2], in which it was found that to avoid gag reflex, microcurrent electrical stimulation can be applied to patients on the auricular and Hegus acupoints. It can be deduced from the two studies that possibly there is another way of reducing the severity of a gag reflex.

Agrawal et al. [2] determined that Point B Hegus Li4 was better than Point A auricular in order to suppress the gag reflex. On the other hand, our research suggested that the difference between the GPI of the normal and modified earplugs is not statistically significant. This mismatch can be achieved from the different methods each investigation adopted.

Samaleti and Jawdekar [9] compared the "earplug and temporal tap technique" with the conventional distraction method for finding out which may reduce the gag reflex during children's maxillary impression-making. They found that though the earplug and temporal tap technique improved the experience, they did not reduce the gag reflex. Contrarily, our study shows that the effectiveness of GPI is more prominent in reducing the severity than GSI. This difference might be because of different age groups and methodologies adopted by the researches.

Rosted et al. [10] evaluated the effect of the acupuncture point CV-24 in alleviating a severe gag reflex during dental procedures with an upper alginate imprint. They had presented a statistically significant reduction in gag reflex scores in all three phases of the process of impression-taking. Similarly, Fiske and Dickinson [11] reported that out of the total 10 cases, eight cases had demonstrated complete suppression of gag reflex through ear acupuncture and two cases, partial suppression. These reports support our study and at the same time suggest that various modes can be used to reduce the severity of the gag reflex.

Akarslan and Biçer [12] investigated the usefulness of the Gagging Problem Assessment (GPA) questionnaire in the assessment of patient sensitivity to dental operations. They observed that the GPA scores can predict the patient's sensitivity to dental treatments. In the present study, no such GPA questionnaire was used; however, questionnaires of a similar nature may be implemented to assess gag reflex susceptibility among patients in the future. 

Research has consistently shown that gagging is a natural defense mechanism to prevent foreign objects from entering the upper respiratory tract [13]. However, severe gagging can occur during dental and prosthodontic procedures, leading to difficulties for both clinicians and patients and compromising treatment quality [13]. The type of soft palate has been found to be associated with gag reflex, with females being more prone to severe gagging [14]. An abnormal gag reflex can have significant social and oral health implications, as individuals may avoid dental treatment due to anxiety or discomfort [15].

Various methods have been proposed to manage the gag reflex, with distraction techniques, nitrous oxide, and low-level laser therapy being shown to be effective [16]. Sedation techniques, such as propofol administration, have also been investigated for gag reflex suppression [17]. The importance of managing gag reflex has been highlighted, particularly during the COVID-19 pandemic, with various pharmacological and non-pharmacological interventions being suggested [18].

A hypersensitive gag reflex can significantly impact dental treatment outcomes, and effective management strategies are essential to ensure successful clinical stages [19]. Identifying the cause and severity of the gag reflex and using various modalities, such as distraction techniques, nitrous oxide, and low-level laser therapy, can help make patients more comfortable and cope with dental treatment [20].

There are various limitations of this study that have to be taken into account while interpreting the results. First, the sample size was small and may not have given enough power to detect clinically statistically significant differences between the two groups concerning the conventional and modified earplugs. Second, this study only examined the effectiveness of earplugs in decreasing gag reflex severity but did not assess other outcomes that could be realized by using this device, such as patients' satisfaction or levels of anxiety. It also did not try to eliminate any confounding variable effects that might have had an impact on the results, such as anxiety or previous experiences related to making an impression. Future studies should address these limitations by recruiting larger, more diverse samples and measuring a broader range of outcomes.

## Conclusions

Our findings lend credence to the fact that the modified earplugs are an effective adjunct to music therapy in reducing gag reflex severity compared to the conventional earplugs and thus represent important implications for the management of gag reflex in completely edentulous patients, pointing out advantages that could be derived from a multifaceted approach to this common problem. Further studies are required to have a full understanding of the design and optimal functionality of earplugs for gag reflex reduction; therefore, this intervention may be explored in other patient populations as well.
